# Reply to “Comment on Fitria et al. ‘Environmental and Occupational Risk Factors Associated with Chronic Kidney Disease of Unknown Etiology in West Javanese Rice Farmers, Indonesia’ *Int. J. Environ. Res. Public Health*, 2020, 17, 4521”

**DOI:** 10.3390/ijerph17197273

**Published:** 2020-10-05

**Authors:** Laila Fitria, Nurhayati Adnan Prihartono, Doni Hikmat Ramdhan, Susan Woskie

**Affiliations:** 1 Department of Environmental Health, Faculty of Public Health, University of Indonesia, Depok 16424, Indonesia; 2 Department of Epidemiology, Faculty of Public Health, University of Indonesia, Depok 16424, Indonesia; nurhayati-a@ui.ac.id; 3 Department of Occupational Health and Safety, Faculty of Public Health, University of Indonesia, Depok 16424, Indonesia; doni@ui.ac.id; 4 Department of Public Health, University of Massachusetts Lowell, One University Ave, Lowell, MA 01854-2867, USA; Susan_Woskie@uml.edu

**Keywords:** CKD, CKDu, AKD, WBGT

## Abstract

Examining the WBGT (Wet Bulb Globe Temperature) profiles in the two study locations was intended to show temperature differences during the week of the study. Information obtained from the weather stations that provide contextual long-term information on heat and humidity also showed temperature differences. The average measured temperature and humidity in the past year from each of these weather stations show an average heat index of 22 °C in Bogor and an average heat index of 32 °C in Karawang. Interpretation of the chronic kidney disease (CKD) and chronic kidney disease of unknown etiology (CKDu) findings was more complicated because we also found that farmers in our two locations reported differences in the use of mechanization in their farming, presumably impacting their workloads.

Thank you for the opportunity to provide a response to the comment submitted by Jakobsson et al. [1] on our paper “Environmental and Occupational Risk Factors Associated with Chronic Kidney Disease of Unknown Etiology in West Javanese Rice Farmers, Indonesia” [2].

This was our first study examining chronic kidney disease (CKD) and chronic kidney disease of unknown etiology (CKDu). We appreciate the comments provided by these highly esteemed CKD researchers, and hope to incorporate their recommendations in future work.

Our study focused on farmers as a high risk population, based on previously published studies [3,4,5,6,7]. We examined altitude as a risk factor for CKD, as we expected the two selected locations to have different WBGT (Wet Bulb Globe Temperature) profiles based on their altitude. The WBGT results are being reported in a paired manuscript that includes information on heat-related symptoms and musculoskeletal disorders among these two groups of farmers [8]. Figure 1 summarizes the measurements made hourly over the week.

We agreed that contextual long-term information on heat and humidity that is available from existing weather stations would provide additional information on the potential for heat stress among workers in these areas. In our country, not all of the regencies have a weather station. For our study, the Bogor Regency has a weather station near the village (14 km). However, for our study location in Karawang Regency, the nearest weather station is in East Jakarta (54 km). From the available data, we calculated the average measured temperature and humidity in the past year from each of these weather stations [9]. From the weather station in Bogor Regency, the average temperature and humidity were 21.5 °C and 85.9% for an average heat index of 22 °C. Form the weather station in East Jakarta, the average temperature and humidity were 28 °C and 77% for a heat index of 32 °C. While these heat index averages are classified as “caution” (“fatigue possible with prolonged exposure and/or physical activity”), exposure to direct sunlight can add up to 9.4 °C to the heat index, resulting in a “danger” categorization for Karawang (“heat cramps or heat exhaustion likely, heat stroke possible with prolonged exposure and/or physical activity”) [10]. However, in addition to WBGT differences, we also found that farmers in our two locations reported differences in the use of mechanization in their farming, presumably impacting their workloads. This made interpretation of the CKD findings more complex.

As requested, we are providing additional data on the distribution of renal function by presenting a more detailed breakdown of eGFR (estimated Glomerular Filtration Rate) and proteinuria results in Table 1.

Thank you for your suggestions regarding enhanced reporting of our data on renal function among Indonesian rice farmers. As you note, little work has been done on this issue in Southeast Asia, yet we are an equatorial climate with a large agricultural workforce who may be at significant risk of developing CKD.

## Figures and Tables

**Figure 1:**
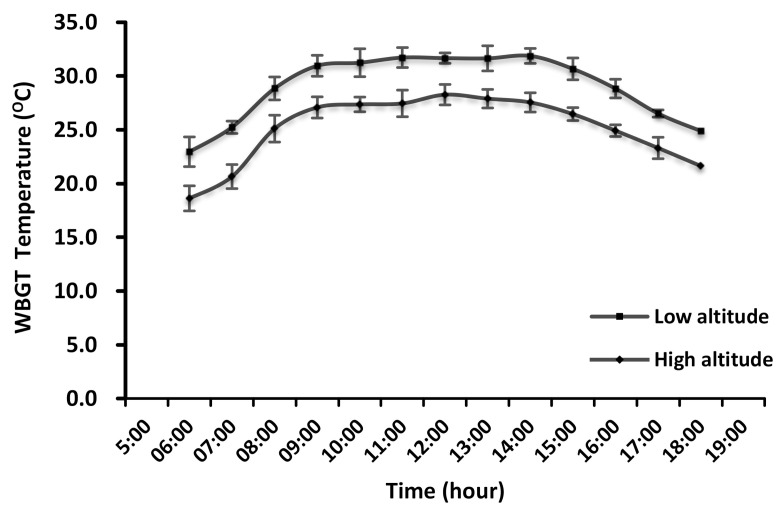
Average WBGT (Wet Bulb Globe Temperature) hourly measurements in a low-altitude area (Karawang Regency) and a high-altitude area (Bogor Regency).

**Table 1:** Distribution of renal parameters of male farmers in Karawang Regency and Bogor Regency, West Java ^1^**.**

Renal Parameters	Karawang (n = 186)	Bogor (n = 168)	Total
	n (%)	n (%)	n (%)
Proteinuria			
Positive +4 (2000+ mg/dL)	1 (0.5)	0 (0)	1 (0.3)
Positive +3 (300 mg/dL)	1 (0.5)	0 (0)	1 (0.3)
Positive +2 (100 mg/dL)	5 (2.7)	8 (4.8)	13 (3.7)
Positive +1 (30 mg/dL)	31 (16.7)	6 (3.6)	37 (10.5)
Negative or Trace	148 (79.6)	154 (91.7)	302 (85.3)
eGFR CKD-MDRD^2^ (ml/min/1.73 m^2^)			
<60	5 (2.7)	7 (4.2)	12 (3.4)
≥60	181 (97.3)	161 (95.8)	342 (96.6)
eGFR CKD-MDRD (ml/min/1.73 m^2^)			
<15	0 (0)	0 (0)	0 (0)
15–29	1 (0.5)	1 (0.6)	2 (0.6)
30–59	4 (2.2)	6 (3.6)	10 (2.8)
60–89	27 (14.5)	42 (25.9)	69 (19.5)
≥90	154 (82.8)	119 (70.8)	273 (77.1)
AKD ^3^ grouping			
eGFR < 90 + proteinuria < 2	28 (15.1)	45 (26.8)	73 (20.6)
eGFR < 90 + proteinuria < 2 + exclusion ^2^	20 (10.8)	32 (19.0)	52 (14.7)
eGFR < 90 + proteinuria ≥ 2	4 (2.2)	4 (2.4)	8 (2.3)
eGFR < 90 + proteinuria ≥ 2 + exclusion ^2^	3 (1.6)	2 (1.2)	5 (1.4)

^1^ Karawang: location with low altitude and high WBGT and more modern mechanized farming. Bogor: location with high altitude and low WBGT and more traditional non-mechanized farming. ^2^ Excluded subjects with diabetes based on blood sugar concentration (>200 mg/dL considered as diabetes) or self-reported diabetes or hypertension based on blood pressure (systole/diastole ≥ 140/≥ 90 considered as hypertension) or self-reported hypertension with medication or self-reported urethritis, gout and kidney stone. ^2^ eGFR CKD-MDRD: estimated Glomerular Filtration Rate, Chronic Kidney Disease—Modification of Diet in Renal Disease. ^3^ AKD: Acute Kidney Disease.
